# COVID-19 and Personality: A Cross-Sectional Multicenter Study of the Relationship Between Personality Factors and COVID-19-Related Impacts, Concerns, and Behaviors

**DOI:** 10.3389/fpsyt.2021.608730

**Published:** 2021-02-26

**Authors:** Mahmoud K. AL-Omiri, Ibrahim A. Alzoubi, Abdullah A. Al Nazeh, Abdallah K. Alomiri, Mohannad N. Maswady, Edward Lynch

**Affiliations:** ^1^Department of Prosthodontics, School of Dentistry, University of Jordan, Amman, Jordan; ^2^Department of Prosthodontics, The City of London Dental School, Canada Water, London, United Kingdom; ^3^Department of Preventive Dentistry, College of Dentistry, Jouf University, Sakaka, Saudi Arabia; ^4^Department of Paediatric Dentistry and Orthodontics, College of Dentistry, King Khalid University, Asir–Abha, Saudi Arabia; ^5^Faculty of Allied Medical Sciences, Audiology and Speech Pathology Department, Al Ahliyya Amman University, Amman, Jordan; ^6^Department of Information Technology, The International Academy, Amman, Jordan; ^7^School of Dental Medicine, University of Nevada Las Vegas (UNLV), Las Vegas, NV, United States

**Keywords:** COVID-19, SARS-CoV-2, NEO-FFI, personality, concerns, psychology

## Abstract

**Background:** This cross-sectional observational study aimed to evaluate coronavirus disease 2019 (COVID-19)-related precautions to avoid COVID-19 infection, distress and behavioral changes, fears and concerns, and effects on opinions and beliefs among participants from different backgrounds and also to identify the relationships between personality factors and COVID-19-related changes and impacts.

**Methods:** One thousand and three hundred nineteen participants (744 females and 575 males, mean age ± SD = 32.7 ± 11.6 years) completed a three-section survey collecting data regarding demographic information, personality factors [using the NEO Five-Factor Inventory (NEO-FFI)], and COVID-19-related issues (using the VAS scale).

**Findings:** COVID-19 was associated with changes related to precautions to avoid COVID-19 infection, distress and behavioral changes, fears and concerns, and effects on opinions and beliefs (*P* < 0.05). Higher neuroticism scores were associated with more negative COVID-19-related changes and impacts (*P* < 0.05). Higher extraversion, agreeableness, and conscientiousness scores were associated with more acceptance of COVID-19 containment measures as well as less COVID-19-related changes and impacts (*P* < 0.05).

**Conclusions:** Personality factors were associated with COVID-19-related impacts. These findings demonstrate the importance of the relationship between personality factors and COVID-19-related changes.

## Introduction

Many countries imposed complete or partial curfews and applied measures to enhance social distancing in attempts to control the coronavirus disease 2019 (COVID-19) pandemic.

The fast large-scale spreading of COVID-19 and the associated containment measures have resulted in extensive changes in people's life styles and posed serious challenges to healthcare systems as well as the economy (1–4). Fear and anxiety have spread among the public as well as healthcare workers during the struggle against this disease (4–6). COVID-19 and associated containment measures could have been accompanied with various concerns and might affect the mental and psychological well-being of individuals (3, 6–8). This could have been aggravated by the so called “infomedia” which spread information using various social media platforms causing an “infodemic” that enhanced fear and uncertainty due to the disease (6, 9, 10).

The effects of COVID-19 on personality profiles and psychological traits are still not completely understood. In China, it was reported that immediate psychological reactions during early phases of COVID-19 have included stress, depression, and anxiety (11). Similarly, negative psychological impacts of COVID-19 including avoidance were reported among the Spanish population (8). Also, psychological distress, anger, fear, hopelessness, and traumatic feelings were reported among the Czech population (6). In addition, supernatural causal beliefs and dysfunctional personality domains (including negative affectivity and detachment) were associated with more emotional problems among Italian community house residents (12).

More negative emotions (higher levels of depression, anxiety, indignation, and sensitivity to social risks) and less positive emotions (lower levels of happiness and life satisfaction) were reported following the declaration of the COVID-19 epidemic (13).

Concerns regarding inability to control the infection, fear of getting infected, and professional and financial expectations were related to anxiety, psychological distress, and higher risks of moral injury (14–16). Healthcare professionals, persons of younger age, and people thinking for a long time concerning the infection were also associated with higher risk of anxiety, depression, and insomnia (4, 17, 18). This could be due to fear from getting infected, fear of inability to control the infection, lack of rest, increased working time, and concerns regarding own health, family health, isolation, virus transmission, and lack of resources (4, 5, 19).

Previous literature highlighted that quarantine might be associated with fear, stress, depression, anger, low self-esteem, mood disorders, lack of self-control, psychological distress, posttraumatic stress symptoms, insomnia, confusion, nervousness, irritability, and sadness (1, 2, 7, 20). The psychological consequences of quarantine might result due to fear from getting infected, extended quarantine periods, frustration, stigma, dullness, misinformation, financial burden, and insufficient supplies and resources (1, 2, 7). Also, among self-isolated individuals, decreased levels of social capital were accompanied with increased stress, anxiety, and insomnia (20).

However, a recent meta-analysis review concluded that long-term psychiatric disorders or neuropsychiatric complications are rare following coronavirus infections (21). Furthermore, a national Chinese survey reported that psychological distress was significantly reduced following quarantine, implementing social distancing measures, education of the public, and making medical resources available nationwide (22). Also, some researchers found that anxiety had no significant relationships with behavior changes or measures used by the public to prevent infection among a Chinese population (18).

Some previous studies have inspected the relationship between COVID-19-associated concerns and behaviors and the Five-Factor Model of Personality (FFM) traits. Lower levels of extroversion and higher levels of conscientiousness were accompanied with better commitment to apply handwashing and social distancing as containment measures against COVID-19 among a Brazilian population (23). Higher openness was associated with better hygiene practice, while lower extraversion and higher neuroticism, agreeableness, and conscientiousness were accompanied with better hygiene and social distancing among a US population (24). Also, Aschwanden et al. found that higher neuroticism was associated with more pessimistic duration estimates, more concerns, and fewer precautions in response to COVID-19 in the USA (25). They also found that higher conscientiousness was associated with more precautions, and higher extraversion was associated with more optimistic duration estimates. Furthermore, lower neuroticism and higher openness were associated with improved sanitation and better social distancing in the USA (26). In addition, lower neuroticism and higher extraversion, agreeableness, openness, and conscientiousness were associated with better commitment to apply formal COVID-19 instructions (27). Moreover, better social distancing was related to higher extraversion, and better sanitation and handwashing were related to higher extraversion and conscientiousness among a USA population (28). Also, less negative impacts on well-being were associated with higher openness and conscientiousness (29). Meanwhile, more negative impacts on daily living were associated with higher neuroticism during the pandemic in Germany (30). Also, better commitment to social distancing was related to higher neuroticism and conscientiousness in Qatar (31). In addition, higher agreeableness was associated with better commitment to governmental measures against COVID-19 among a German student population (32). Finally, lower extraversion and higher openness, agreeableness, and conscientiousness were associated with more compliance with measures to prevent COVID-19 transmission in Japan (33). However, previous studies did not compare different countries in this regard.

The literature lacks multicenter investigations on the relationship between FFM personality individual differences and concerns related to the disease itself and the associated containment measures. Also, research is scarce on comprehensive assessment of what personality factors might affect the compliance with disease containment and prevention measures as well as disease impacts on different individuals, and further research is required in this regard.

This manuscript aimed to evaluate personality factors [*via* NEO Five-Factor Inventory (NEO-FFI) personality questionnaire (34)] and COVID-19-related precautions to avoid and contain COVID-19 infection, distress and behavioral changes, fears and concerns, and effects on opinions and beliefs among participants from different backgrounds. Also, it aimed to identify the relationship between personality factors and COVID-19 impacts on precautions to avoid COVID-19 infection, distress and behavioral changes, fears and concerns, and effects on opinions and beliefs.

The null hypothesis for this study was that COVID-19 does not impact precautions to avoid infection and is not associated with distress and behavioral changes, fears and concerns, and effects on opinions and beliefs among participants from different backgrounds. Also, personality factors and individual differences are not related to COVID-19-related precautions to avoid COVID-19 infection, distress and behavioral changes, fears and concerns, and effects on opinions and beliefs.

## Methods

### Study Design, Study Instrument, and Population

This cross-sectional observational investigation was conducted between 23rd and 30th May 2020 utilizing an online Google Forms survey tool. It was carried out following the ethical standards of the World Medical Association Declaration of Helsinki. This study was ethically approved by the Institutional Review Board, University of Jordan, Jordan (Reference number: 223/2020/19/31). The study was reported following STROBE statement and guidelines.

The participants were provided with the detailed explanation of the study and were requested to consent to participate before going further and complete the survey. The survey was completely anonymous, participants' identity was not identified, and the collected data was confidential. An email address was provided at the start of the survey for the participants to contact if they have any concerns or if they request any explanation or information regarding this study and the survey involved. The survey was designed to allow one response per participant.

The participants were invited to participate and were included in the study if they were 18 years old or above and able to understand and complete the survey. Participants included in the mailing lists at multiple centers in Jordan (University of Jordan and Jordan University of Science and Technology), KSA (King Khalid University), UK (BAIRD Academy), and Palestine (Al Quds University and Islamic University Gaza) were eligible to be recruited into this study. Participants on mailing lists at each center were selected following a multistage random sampling process according to gender to secure a representative sample. Five hundred participants (250 males and 250 females) were selected and invited from each region. The survey link was first sent to the participants *via* email, then it was sent through WhatsApp groups and social media websites Twitter, Instagram, and Facebook to encourage the maximum number of responses.

The survey consisted of three sections; the participants were provided with explanation of the survey sections and how to complete the survey. The first section of the survey included an explanation of the study as well as questions related to demographic data including age, gender, marital status, income, occupation, living area, level of education, specialty, student status, source of information regarding COVID-19, cross infection control general training, and received training to combat COVID-19. Meanwhile, the second section of the survey included the NEO-FFI personality questionnaire (34), which comprised 60 statements and was used to assess participants' personality factors including neuroticism, extraversion, openness, agreeableness, and conscientiousness. The third section included evaluation of COVID-19-related impacts on participants' concerns, fears, behaviors, opinions, everyday living, and habits utilizing 50 statements that were answered on a Visual Analog Scale (VAS) scale from 0 to 10; 0 means completely disagree with the statement and 10 means completely agree with the statement.

Primary outcome measures were personality dimensions (*via* NEO-FFI personality scores) and COVID-19-related impacts on participants' concerns, worries, fears, behaviors, everyday living, and habits (*via* VAS scores). COVID-19-related impacts were presented under four major categories including precautions to avoid COVID-19 infection, distress and behavioral changes, fears and concerns, and effects on opinions and beliefs.

### Statistical Analysis

The SPSS software (IBM SPSS Statistics v19.0; IBM Corp., USA) was utilized for statistical data analysis in this study. Frequencies and descriptive statistics of various variables in this study were identified and tabulated. Test for data normal distribution was conducted using the Kolmogorov–Smirnov test. Correlations between different variables (including demographic variables, NEO-FFI scores, and VAS scores of COVID-19-related impacts) were initially assessed using Spearman's correlation tests to identify raw results before considering the confounding effects of demographic variables (gender, age, level of education, and race). NEO-FFI personality scores as well as COVID-19-related changes were compared between groups based on different demographic variables and were calculated using the Mann–Whitney *U*-test (for comparisons of two groups) and Kruskal–Wallis test (for comparisons of more than two groups). Spearman's correlation test was carried out to identify the associations between NEO-FFI scores and VAS scores of COVID-19-related impacts. Multiple regression analysis was used to predict COVID-19-related changes and impacts utilizing NEO-FFI personality scores. Significant statistical outcomes were set at α values ≤ 0.05 and 95% confidence intervals. Statistics were two-tailed unless otherwise mentioned.

Confounding effects of demographic variables were considered during the statistical analysis in this study. Stratification of data analysis by gender and country (race) was carried out to identify differences in NEO-FFI scores as well as VAS scores of COVID-19-related impacts. Also, hierarchical multiple regression analysis was conducted in order to control the confounding effects of demographic variables (gender, age, level of education, and race) on the relationship between NEO-FFI personality scores and VAS scores of COVID-19-related impacts. The hierarchical multiple regression analysis was conducted including the demographic variables in the first block of independent variables and the NEO-FFI scores in the second block of independent variables to account for the confounding effects of the demographic variables on the contribution of NEO-FFI scores toward the VAS scores of COVID-19-related impacts (i.e., ability of NEO-FFI scores to predict VAS scores of COVID-19-related impacts).

Sample size calculation for this inquiry was performed utilizing a computer software (G^*^Power, version 3.1.9.7; Heinrich-Heine University). The sample size was estimated using *a priori* power analysis *via F* test for linear multiple regression fixed model with *R*^2^ increase design in the software. The calculated sample size was equal to 955 participants considering statistical power (1 – β) of 0.95, significance level (α) of 0.05, and effect size of 0.02. Extra participants were recruited to compensate for the participants who would be excluded if they returned incomplete survey. One thousand and three hundred thirty-one responses were obtained from the 2,000 invited participants with a response rate of 66.55%. After assessment of responses, 12 participants were excluded from the study because their responses were incomplete (dropout rate is 0.90%); therefore, the total number of participants included in this study was 1,319 (65.95% of the invited participants).

## Results

### Distribution of Demographic Data, NEO-FFI Scores, and COVID-19-Related Impacts Among the Study Participants

In total, 1,319 participants (744 females and 575 males, mean age = age range: 18–75 years old) were included in this study and the data collected from them were interpreted and analyzed. Participants' age ranged between 18 and 75 years old (mean age ± SD = 32.7 ± 11.6 years for the whole study sample, 30.6 ± 10.4 years for females, and 35.5 ± 12.6 years for males). Frequencies and distribution of demographic variables among the study participants are presented in Table 1.

**Table 1:** Distribution of demographic variables among participants according to gender.

**Demographic variable**	**Number of participants (percentage of participants)**		
	**Total**	**Female**	**Male**
Gender	1,319 (100%)	744 (56.4%)	575 (43.6%)
Marital status: Single	681 (51.6)	439 (59.0)	242 (42.1)
Married	638 (48.4)	305 (41.0)	333 (57.9)
Living area: City	1,077 (81.7)	609 (81.9)	468 (81.4)
Town	242 (18.3)	135 (18.1)	107 (18.6)
Student: Yes	499 (37.8)	313 (42.1)	186 (32.3)
No	820 (62.2)	431 (57.9)	389 (67.7)
Education: High school or equivalent	189 (14.3)	106 (14.2)	83 (14.4)
College diploma or equivalent	94 (7.1)	58 (7.8)	36 (6.3)
Bachelor's degree	772 (58.5)	489 (65.7)	283 (49.2)
MSc/MDSc or equivalent	139 (10.5)	57 (7.7)	82 (14.3)
PhD or equivalent	125 (9.5)	34 (4.6)	91 (15.8)
Specialty: Medical field	734 (55.6)	393 (52.8)	341 (59.3)
None medical field	585 (44.4)	351 (47.2)	234 (40.7)

Table 2 presents the frequencies and distribution of participants who scored very high, high, average, low, and very low on NEO-FFI dimension scores in comparison with the NEO-FFI manual norms in each country and for each gender. Participants from different countries were found to score differently in comparison with the NEO-FFI manual norms regarding openness (Kruskal–Wallis test, χ^2^ = 30.425, *df* = 3, *P* ≤ 0.0001), agreeableness (χ^2^ = 11.256, *df* = 3, *P* = 0.010), and conscientiousness (χ^2^ = 20.436, *df* = 3, *P* ≤ 0.0001). However, no differences were found regarding neuroticism (χ^2^ = 4.404, *df* = 3, *P* = 0.221) and extraversion (χ^2^ = 1.093, *df* = 3, *P* = 0.779). Table 2 shows the difference between genders within each country in this regard.

**Table 2:** Distribution of NEO-FFI personality dimension scores among participants according to gender (*N* = 1,319; 575 males and 744 females).

	^*^Number of participants who reported (percent of participants)															**MWU**	***Z***	***P***
**Pers. dim**.	**Very high score**			**High score**			**Average score**			**Low score**			**Very low score**					
	**M**	**F**	**T**	**M**	**F**	**T**	**M**	**F**	**T**	**M**	**F**	**T**	**M**	**F**	**T**			
*N* All	36 (6.3)	62 (8.3)	98 (7.4)	172 (29.9)	197 (26.5)	369 (28.0)	253 (44.0)	296 (39.8)	549 (41.6)	95 (16.5)	173 (23.3)	268 (20.3)	19 (3.3)	16 (2.2)	35 (2.7)			
Jordan	12	14	26	35	45	80	71	91	162	21	42	63	6	2	8	13,530.0	−0.642	0.521
KSA	10	18	28	49	53	102	69	68	137	18	39	57	2	5	7	12,729.5	−0.993	0.321
Palestine	6	10	16	46	54	100	56	76	132	25	44	69	6	4	10	12,610.0	−0.570	0.569
UK	8	20	28	42	45	87	57	61	118	31	48	79	5	5	10	12,756.0	−0.054	0.957
E All	29 (5.0)	11 (1.5)	40 (3.0)	202 (35.1)	148 (19.9)	350 (26.5)	245 (42.6)	409 (55.0)	654 (49.6)	84 (14.6)	135 (18.1)	219 (16.6)	15 (2.6)	41 (5.5)	56 (4.2)			
Jordan	4	5	9	45	44	89	66	101	167	24	34	58	6	10	16	12,934.0	−1.368	0.171
KSA	4	1	5	64	33	97	54	105	159	20	35	55	6	9	15	9,994.0	−4.422	^**^
Palestine	9	2	11	47	31	78	63	109	172	18	34	52	2	12	14	9,570.0	−4.522	^**^
UK	12	3	15	46	40	86	62	94	156	22	32	54	1	10	11	10,196.0	−3.376	0.001
O All	10 (1.7)	10 (1.3)	20 (1.5)	48 (8.3)	73 (9.8)	121 (9.2)	245 (42.6)	334 (44.9)	579 (43.9)	205 (35.7)	279 (37.5)	484 (36.7)	67 (11.7)	48 (6.5)	115 (8.7)			
Jordan	0	2	2	5	15	20	65	82	147	52	82	134	23	13	36	12,720.0	−1.629	0.103
KSA	0	2	2	10	19	29	49	84	133	68	65	133	21	13	34	10,825.5	−3.367	0.001
Palestine	0	2	2	13	14	27	64	87	151	47	72	119	15	13	28	12,951.5	−0.147	0.883
UK	10	4	14	20	25	45	67	81	148	38	60	98	8	9	17	11,722.5	−1.388	0.165
A All	5 (0.9)	0 (0.0)	5 (0.4)	18 (3.1)	7 (0.9)	25 (1.9)	88 (15.3)	92 (12.4)	180 (13.6)	241 (41.9)	301 (40.5)	542 (41.1)	223 (38.8)	344 (46.2)	567 (43.0)			
Jordan	0	0	0	3	0	3	21	21	42	51	80	131	70	93	163	13,656.5	−0.502	0.615
KSA	0	0	0	0	1	1	19	24	43	73	78	151	56	80	136	12,927.0	−0.778	0.436
Palestine	1	0	1	3	2	5	21	20	41	64	74	138	50	92	142	11,131.0	−2.497	0.013
UK	4	0	4	12	4	16	27	27	54	53	69	122	47	79	126	10,524.5	−2.919	0.004
C All	36 (6.3)	41 (5.5)	77 (5.8)	89 (15.5)	114 (15.3)	203 (15.4)	246 (42.8)	311 (41.8)	557 (42.2)	155 (27.0)	173 (23.3)	328 (24.9)	49 (8.5)	105 (14.1)	154 (11.7)			
Jordan	14	9	23	32	36	68	57	94	151	26	37	63	16	18	34	13,226.0	−0.993	0.321
KSA	7	11	18	11	27	38	66	71	137	44	47	91	20	27	47	12,705.0	−1.017	0.309
Palestine	6	14	20	28	28	56	59	77	136	43	45	88	3	24	27	12,188.0	−1.093	0.274
UK	9	7	16	18	23	41	64	69	133	42	44	86	10	36	46	11,184.5	−2.044	0.041

*Pers. dim., personality dimension; All, all; N, neuroticism; E, extraversion; O, openness; A, agreeableness; C, conscientiousness; T, total; M, males; F, females; MWU, Mann–Whitney U-test statistic; Z, Z statistic; P, two-tailed significance probability value*.

* *In comparison to the NEO Five-Factor Inventory manual norms*.

** *P < 0.0001*.

Table 3 presents the means, standard deviations, confidence intervals, and gender differences of NEO-FFI dimension scores within each country and for each gender. Considering the whole study sample, females scored lower on extraversion (Mann–Whitney *U* = 181,369.0, *Z* = −4.753, *P* < 0.0001, 95% CI = 28.00–28.53) and higher on neuroticism (Mann–Whitney *U* = 173,989.5, *Z* = −5.824, *P* < 0.0001, 95% CI = 20.21–20.99) and agreeableness (Mann–Whitney *U* = 186,342.5, *Z* = −4.027, *P* < 0.0001, 95% CI = 26.21–26.69). Meanwhile, no gender differences were found regarding openness (Mann–Whitney *U* = 207,678.5, *Z* = −0.909, *P* = 0.363, 95% CI = 24.04–24.57) and conscientiousness (Mann–Whitney *U* = 204,452.5, *Z* = −1.379, *P* = 0.168, 95% CI = 32.74–33.44) (Table 3). Table 4 shows the sample differences according to student status as well as working/studying within a medical field.

**Table 3:** Distribution of NEO-FFI personality dimension scores among participants in each country and differences between genders (*N* = 1,319; 575 males and 744 females).

**Personality dimension**	**Mean score (±SD)**			**95% confidence intervals**			**MWU**	***Z***	***P***
	**Males**	**Females**	**Total**	**Males**	**Females**	**Total**			
Neuroticism: All samples	19.17 (6.80)	21.70 (7.43)	20.60 (7.27)	18.62–19.73	21.17–22.24	20.21–20.99	173989.5	−5.824	<0.0001
Jordan	19.23 (6.95)	21.49 (6.95)	20.53 (7.03)	18.09–20.37	20.51–22.48	19.77–21.28	11519.5	−2.855	0.004
KSA	20.01 (6.61)	22.07 (7.736)	21.15 (7.32)	18.93–21.08	20.94–23.19	20.35–21.94	11268.5	−2.629	0.009
Palestine	18.88 (6.77)	21.61 (7.00)	20.45 (7.02)	17.74–20.01	20.60–22.61	19.68–21.21	10316.0	−3.258	0.001
UK	18.54 (6.86)	21.66 (8.07)	20.28 (7.70)	17.40–19.67	20.47–22.85	19.43–21.12	10232.5	−3.094	0.002
Extraversion: All samples	28.97 (4.79)	27.72 (4.96)	28.26 (4.92)	28.57–29.36	27.36–28.08	28.00–28.53	181369.0	−4.753	<0.0001
Jordan	28.25 (4.94)	28.07 (5.06)	28.15 (5.00)	27.44–29.06	27.36–28.79	27.61–28.68	13669.0	−0.445	0.657
KSA	28.82 (4.90)	27.63 (4.80)	28.16 (4.88)	28.03–29.62	26.93–28.33	27.64–28.69	11154.0	−2.765	0.006
Palestine	29.40 (4.41)	27.38 (4.83)	28.24 (4.75)	28.66–30.13	26.69–28.08	27.72–28.76	10028.0	−3.604	<0.0001
UK	29.43 (4.82)	27.78 (5.14)	28.51 (5.06)	28.63–30.22	27.02–28.54	27.96–29.07	10566.0	−2.697	0.007
Openness: All samples	24.17 (4.93)	24.41 (5.01)	24.30 (4.97)	23.77–24.58	24.05–24.77	24.04–24.57	207678.5	−0.909	0.363
Jordan	23.23 (4.24)	23.77 (4.69)	23.54 (4.51)	22.53–23.92	23.11–24.44	23.06–24.02	13549.5	−0.579	0.563
KSA	22.80 (4.18)	24.52 (5.11)	23.76 (4.79)	22.13–23.48	23.78–25.27	23.24–24.27	10655.5	−3.343	0.001
Palestine	24.17 (4.73)	24.17 (4.90)	24.17 (4.82)	23.37–24.96	23.46–24.88	23.64–24.69	13032.5	−0.040	0.968
UK	26.55 (5.61)	25.22 (5.25)	25.81 (5.45)	25.62–27.48	24.45–26.00	25.22–26.41	10917.5	−2.271	0.023
Agreeableness: All samples	25.99 (4.62)	26.81 (4.31)	26.45 (4.46)	25.61–26.37	26.50–27.12	26.21–26.69	186342.5	−4.027	<0.0001
Jordan	25.10 (4.39)	26.52 (4.02)	25.91 (4.23)	24.38–25.82	25.95–27.09	25.46–26.37	11078.0	−3.355	0.001
KSA	25.80 (4.18)	26.95 (4.39)	26.44 (4.33)	25.12–26.48	26.31–27.59	25.97–26.91	11419.5	−2.460	0.014
Palestine	25.91 (4.34)	26.68 (3.93)	26.35 (4.12)	25.19–26.64	26.11–27.24	25.90–26.80	11697.0	−1.625	0.104
UK	27.17 (5.29)	27.12 (4.90)	27.14 (5.07)	26.29–28.04	26.39–27.84	26.58–27.70	12206.0	−0.715	0.474
Conscientiousness: All samples	33.00 (6.34)	33.16 (6.65)	33.09 (6.52)	32.48–33.52	32.68–33.63	32.74–33.44	204452.5	−1.379	0.168
Jordan	34.07 (7.01)	34.20 (5.85)	34.14 (6.36)	32.92–35.22	33.37–35.02	33.46–34.82	14056.5	−0.010	0.992
KSA	31.69 (6.00)	33.20 (6.73)	32.53 (6.45)	30.71–32.66	32.22–34.18	31.83–33.22	11388.0	−2.492	0.013
Palestine	33.60 (5.90)	33.48 (6.37)	33.53 (6.16)	32.62–34.59	34.39–33.63	32.86–34.20	12894.5	−0.203	0.839
UK	32.69 (6.18)	31.64 (7.42)	32.11 (6.90)	31.66–33.71	30.55–32.74	31.35–32.86	12438.0	−0.435	0.664

*SD, standard deviation; MWU, Mann–Whitney U-test statistic, Z, Z statistic; P, two-tailed significance probability value*.

**Table 4:** Distribution and differences of NEO-FFI personality dimension scores among study samples according to student status as well as working/studying within the medical field (*N* = 1,319; 575 males and 744 females).

**Personality score**		**Participants are students^$^**					Participants work/study in the medical field^*^				
	**Yes**	**No**	**MWU**	***Z***	***P***	**Yes**	**No**	**MWU**	***Z***	***P***
N	Mean	21.73	19.92	174842.5	−4.438	<0.0001	20.81	20.34	209590.0	−0.744	0.457
	SD	7.38	7.12				7.597	6.83			
	95% CI	21.08–22.37	19.43–20.40				20.26–21.36	19.79–20.90			
E	Mean	28.17	28.32	200930.5	−0.547	0.585	28.08	28.50	206079.0	−1.257	0.209
	SD	5.13	4.79				5.057	4.74			
	95% CI	27.72–28.63	27.99–28.65				27.71–28.44	28.11–28.88			
O	Mean	24.89	23.95	181683.0	−3.422	0.001	24.93	23.52	178949.0	−5.213	<0.0001
	SD	4.89	4.99				5.188	4.57			
	95% CI	24.46–25.32	23.61–24.29				24.56–25.31	23.15–23.89			
A	Mean	25.62	26.96	170758.5	−5.055	<0.0001	26.55	26.34	211193.0	−0.511	0.609
	SD	4.57	4.33				4.718	4.13			
	95% CI	25.21–26.02	26.67–27.26				26.21–26.89	26.00–26.67			
C	Mean	31.33	34.16	151657.0	−7.900	<0.0001	32.40	33.95	184995.5	−4.327	<0.0001
	SD	6.77	6.12				6.719	6.15			
	95% CI	30.73–31.92	33.74–34.58				31.91–32.89	33.45–34.45			

*N, neuroticism; E, extraversion; O, openness; A, agreeableness; C, conscientiousness; SD, standard deviation; 95% CI, 95% confidence intervals; MWU, Mann–Whitney U-test statistic; Z, Z statistic; P, two-tailed significance probability value*.

$ *N = 1,319, 499 students and 820 non-students*.

* *N = 1,319, 585 from the non-medical field and 734 from the medical field*.

Table 5 shows the distribution of participants' VAS scores for different statements that measured COVID-19-related impacts experienced by the participants and the differences between genders in this regard. The impacts of COVID-19 on participants were categorized into four major categories, namely precautions to avoid COVID-19 infection, distress and behavioral changes, fears and concerns, and effects on opinions and beliefs. All participants applied various precautions to avoid COVID-19 infection; however, females washed and disinfected their hands less frequently than males, applied more strict social and physical distancing, and experienced less daily habit changes than males (*P* <0.05) (Table 5). In addition, females experienced more distress and behavioral impacts than males including feeling more stressed, depressed, and worried (*P* < 0.05) (Table 5). Also, males were more concerned and afraid about losing their jobs, while females were more concerned and afraid to be separated from family and that food and necessary things will not be available or be contaminated with the virus (*P* < 0.05) (Table 5). Moreover, females felt that self-isolation and curfews are acceptable practices during the pandemic (*P* < 0.05) (Table 5).

**Table 5:** Distribution of participants' VAS scores for COVID-19-related changes they experienced and differences between genders (*N* = 1,319; 575 males and 744 females).

**COVID-19-related impacts**	**Mean VAS score (±SD)**			**95% CI**		**MWU**	***Z***	***P***
	**Total**	**Female**	**Male**	**Female**	**Male**			
1. Precautions to avoid COVID-19 infection								
Wash and disinfect hands more frequently	8.13 (2.44)	7.96 (2.54)	8.35 (2.28)	7.78–8.14	8.17–8.54	198112.5	−2.458	0.014
Strict social distancing application	8.42 (2.43)	8.52 (2.40)	8.29 (2.47)	8.35–8.70	8.08–8.49	198777.0	−2.441	0.015
Crowded area avoidance	9.05 (1.90)	9.28 (1.70)	8.76 (2.10)	9.16–9.41	8.58–8.93	178958.5	−6.188	<0.0001
Avoidance of contact with people or their pets	8.66 (2.42)	8.79 (2.36)	8.50 (2.48)	8.62–8.96	8.30–8.70	195192.5	−3.168	0.002
Daily habit changes	7.74 (2.99)	7.45 (3.18)	8.11 (2.69)	7.22–7.68	7.89–8.33	193764.5	−3.104	0.002
2. Feeling behavioral changes and distress								
Stress is more	5.27 (3.64)	5.52 (3.66)	4.95 (3.59)	5.26–5.78	4.66–5.25	193853.0	−2.950	0.003
Depression is more	4.73 (3.69)	4.99 (3.79)	4.39 (3.54)	4.72–5.26	4.10–4.68	193692.5	−2.978	0.003
Sad is more	4.82 (3.73)	4.95 (3.79)	4.66 (3.64)	4.68–5.22	4.36–4.96	203510.0	−1.532	0.126
Worry more about the future	5.65 (3.72)	5.87 (3.72)	5.37 (3.70)	5.60–6.14	5.06–5.67	195692.5	−2.685	0.007
Feel more lonely after COVID-19	3.43 (3.76)	3.30 (3.85)	3.59 (3.65)	3.02–3.58	3.29–3.89	200293.0	−2.071	0.038
3. Fears and concerns								
Afraid to get COVID-19	4.95 (3.66)	4.95 (3.72)	4.97 (3.60)	4.68–5.21	4.67–5.26	212443.5	−0.215	0.830
Afraid no healthcare due to shortage of healthcare staff, room, or equipments	4.91 (3.93)	5.06 (4.05)	4.72 (3.77)	4.76–5.35	4.41–5.03	203490.5	−1.544	0.123
Afraid to lose my job	3.72 (3.99)	3.39 (3.98)	4.14 (3.96)	3.10–3.68	3.82–4.47	189979.5	−3.646	<0.0001
Afraid to be separated from my family or beloved ones	5.97 (3.92)	6.34 (3.96)	5.49 (3.83)	6.06–6.62	5.18–5.80	183991.5	−4.479	<0.0001
Afraid food and necessary things will not be available	5.14 (3.76)	5.35 (3.83)	4.87 (3.66)	5.07–5.62	4.57–5.17	198012.5	−2.346	0.019
Worried about contamination of food or water source with COVID-19 virus	6.33 (3.59)	6.55 (3.59)	6.04 (3.57)	6.29–6.81	5.75–6.34	194539.0	−2.880	0.004
Afraid of economic effects on me and my family	6.84 (3.34)	6.88 (3.35)	6.79 (3.32)	6.64–7.12	6.52–7.06	208105.5	−0.864	0.387
Afraid of punishment if one violates or does not comply with authority measures	6.25 (3.72)	6.13 (3.84)	6.42 (3.55)	5.85–6.40	6.13–6.71	207178.5	−1.003	0.316
Worried about safety of myself and my family	6.46 (3.51)	6.74 (3.48)	6.09 (3.52)	6.49–6.99	5.80–6.38	188969.5	−3.711	<0.0001
4. Effects on opinions and beliefs								
Afraid health authorities are not transparent regarding COVID-19	5.02 (3.85)	4.97 (3.94)	5.09 (3.73)	4.69–5.26	4.78–5.39	211077.0	−0.418	0.676
Home self-isolation is acceptable	8.66 (2.33)	8.89 (2.22)	8.35 (2.43)	8.73–9.05	8.15–8.55	180850.5	−5.527	<0.0001
Imposing curfews is acceptable	8.49 (2.51)	8.73 (2.38)	8.18 (2.63)	8.56–8.90	7.97–8.40	183614.5	−4.996	<0.0001
People communication will change and be different after COVID-19	5.47 (3.61)	5.32 (3.68)	5.67 (3.50)	5.05–5.58	5.38–5.95	203098.5	−1.591	0.112

*VAS, Visual Analog Scale; SD, standard deviation; 95% CI, 95% confidence intervals; MWU, Mann–Whitney U-test statistic; Z, Z statistic; P, two-tailed significance probability value*.

Participants from different countries reported significantly different VAS scores of COVID-19 impacts (*P* < 0.05) (Table 6), except for the precautions to avoid COVID-19 infection including the frequency of washing and disinfecting hands, avoidance of crowded areas, avoidance of contact with people and their pets, and changes in daily habits (*P* > 0.05) (Table 6).

**Table 6:** *Post-hoc* test for differences in VAS scores of COVID-19 impacts between participants from different countries in the study (*N* = 1,319, 744 females and 575 males).

**COVID-19 impacts**	**I**	**J**	**Mean dif**.	**Standard error**	***P***	**95% confidence intervals**	
						**Lower bound**	**Upper bound**
1. Precautions to avoid COVID-19 infection							
Wash and disinfect hands more frequently	No significant differences between any two countries						
Strict social distancing application	4	2	0.395	0.190	0.038	0.02	0.77
	4	3	0.389	0.191	0.042	0.01	0.76
Crowded area avoidance	No significant differences between any two countries						
Avoidance of contact with people or their pets	No significant differences between any two countries						
Daily habit changes	No significant differences between any two countries						
2. Feeling behavioral changes and distress							
Stress is more	4	2	0.654	0.284	0.022	0.10	1.21
Depression is more	4	1	0.628	0.287	0.029	0.07	1.19
	4	2	0.810	0.288	0.005	0.24	1.38
Sad is more	4	1	0.617	0.290	0.033	0.05	1.19
	4	3	0.800	0.292	0.006	0.23	1.37
Worry more about the future	4	1	0.897	0.288	0.002	0.33	1.46
	4	2	1.176	0.290	^**^	0.61	1.74
	4	3	0.767	0.291	0.008	0.20	1.34
Feel more lonely after COVID-19	4	1	0.925	0.292	0.002	0.35	1.50
	4	2	0.657	0.294	0.025	0.08	1.23
	4	3	0.851	0.294	0.004	0.27	1.43
3. Fears and concerns							
Afraid to get COVID-19	1	2	−0.613	0.283	0.030	−1.17	−0.06
	1	3	−0.615	0.284	0.030	−1.17	−0.06
	1	4	−0.626	0.285	0.028	−1.18	−0.07
Afraid no healthcare due to shortage of healthcare staff	3	1	0.877	0.301	0.004	0.29	1.47
	3	2	1.398	0.303	^**^	0.80	1.99
	4	1	1.076	0.302	^**^	0.48	1.67
	4	2	1.597	0.304	^**^	1.00	2.19
Afraid to lose my job	3	1	0.629	0.308	0.041	0.03	1.23
	3	2	1.090	0.309	^**^	0.48	1.70
	4	2	0.904	0.311	0.004	0.29	1.51
Afraid to be separated from my family or beloved ones	3	1	0.830	0.302	0.006	0.24	1.42
	3	2	1.091	0.304	^**^	0.49	1.69
	4	2	0.844	0.305	0.006	0.25	1.44
Afraid food and necessary things will not be available	3	1	0.680	0.290	0.019	0.11	1.25
	3	2	1.207	0.291	^**^	0.64	1.78
	4	1	0.591	0.291	0.042	0.02	1.16
	4	2	1.118	0.292	^**^	0.54	1.69
Worried about contamination of food or water source with COVID-19 virus	3	2	0.863	0.279	0.002	0.32	1.41
	3	4	0.971	0.280	0.001	0.42	1.52
Afraid of economic effects on me and my family	2	1	−1.030	0.255	^**^	−1.53	−0.53
	2	3	−1.073	0.258	^**^	−1.58	−0.57
	2	4	−1.283	0.259	^**^	−1.79	−0.77
Afraid of punishment if one violates or does not comply with authority measures	4	1	−0.755	0.287	0.009	−1.32	−0.19
	4	2	−1.209	0.289	^**^	−1.78	−0.64
	4	3	−0.928	0.290	0.001	−1.50	−0.36
4. Effects on opinions and beliefs
Health authorities are not transparent regarding COVID-19	2	1	−1.091	0.291	^**^	−1.66	−0.52
	2	3	−1.648	0.293	^**^	−2.22	−1.07
	4	1	1.259	0.293	^**^	0.69	1.83
	4	2	2.350	0.294	^**^	1.77	2.93
	4	3	0.702	0.295	0.018	0.12	1.28
Home self-isolation is acceptable	2	1	0.893	0.178	^**^	0.54	1.24
	2	3	0.496	0.179	0.006	0.14	0.85
	2	4	0.907	0.180	^**^	0.55	1.26
	3	1	0.398	0.178	0.026	0.05	0.75
	3	4	0.411	0.181	0.023	0.06	0.77
Imposing curfews is acceptable	2	1	0.569	0.192	0.003	0.19	0.95
	2	3	0.511	0.194	0.009	0.13	0.89
	2	4	0.913	0.195	^**^	0.53	1.30
	4	1	−0.345	0.194	0.075	−0.72	0.04
	4	3	−0.403	0.195	0.040	−0.79	−0.02
People communication will change and be different after COVID-19	2	1	−0.969	0.276	^**^	−1.51	−0.43
	2	3	−0.638	0.278	0.022	−1.18	−0.09
	4	1	0.566	0.278	0.042	0.02	1.11
	4	2	1.535	0.279	^**^	0.99	2.08
	4	3	0.896	0.280	0.001	0.35	1.45

*I, first country; J, second country; 1, Jordan; 2, KSA; 3, Palestine; 4, UK; Mean dif., mean difference between country I and J; P, probability value significance calculated using the least significant difference (LSD) post-hoc test*.

** *P <0.0001*.

### Correlations Between Personality Factors and COVID-19-Related Impacts

Table 6 presents the raw correlations between personality factors and COVID-19-related impacts for the total study population as well as for each country in this study. Significant correlations were found between personality factors and COVID-19-related impacts on participants' applied precautions to avoid COVID-19 infection, distress and behavioral changes, fears and concerns, and effects on opinions and beliefs (*P* < 0.05) (Table 6). It was found that conscientiousness and extraversion had more significant relations with COVID-19 impacts on participants' applied precautions to avoid COVID-19 infection. Meanwhile, neuroticism, extraversion, and openness were less involved in this regard (Table 7). Also, neuroticism, extraversion, agreeableness, and conscientiousness were more significantly related with COVID-19 impacts on participants' distress and behaviors. Meanwhile, openness was less involved in this regard (Table 7). In addition, neuroticism and agreeableness had more significant relations with COVID-19 impacts on participants' concerns and fears. However, conscientiousness had less significant correlations than neuroticism and agreeableness; meanwhile, extraversion and openness were the least involved in this regard (Table 7). Finally, neuroticism and agreeableness had more significant relations with COVID-19 impacts on participants' opinions and beliefs. However, conscientiousness had less significant correlations than neuroticism and agreeableness; meanwhile, extraversion and openness were the least involved in this regard (Table 7).

**Table 7:** Correlations between personality factors and COVID-19 impacts among the study population (*N* = 1,319, 744 females and 575 males).

**COVID-19-related impacts**	**Personality factors**									
	**N**		**E**		**O**		**A**		**C**	
	***r***	***P***	***r***	***P***	***r***	***P***	***r***	***P***	***r***	***P***
1. Precautions to avoid COVID-19 infection										
Wash and disinfect hands more frequently: Total	−0.056	0.043	0.152	^**^	−0.009	0.737	0.010	0.711	0.157	^**^
Jordan	−0.115	0.035	0.135	0.013	−0.051	0.346	0.110	0.043	0.149	0.006
KSA	−0.076	0.168	0.181	0.001	−0.025	0.647	−0.041	0.459	0.213	^**^
Palestine	0.021	0.700	0.152	0.006	0.002	0.978	−0.040	0.467	0.176	0.001
UK	−0.079	0.158	0.146	0.009	0.057	0.308	0.015	0.793	0.117	0.035
Strict social distancing application: Total	0.021	0.439	0.059	0.031	0.070	0.011	0.039	0.154	0.118	^**^
Jordan	−0.010	0.853	0.070	0.196	−0.013	0.808	0.051	0.350	0.093	0.088
KSA	0.101	0.067	−0.012	0.826	0.078	0.158	−0.012	0.832	0.200	^**^
Palestine	0.047	0.397	0.046	0.408	0.038	0.497	−0.007	0.897	0.052	0.350
UK	−0.055	0.326	0.132	0.018	0.148	0.008	0.118	0.034	0.150	0.007
Crowded area avoidance: Total	−0.026	0.351	0.064	0.021	0.035	0.208	0.086	0.002	0.166	^**^
Jordan	−0.031	0.570	0.111	0.041	−0.060	0.271	0.089	0.100	0.235	^**^
KSA	−0.057	0.304	0.041	0.455	0.113	0.040	0.097	0.079	0.292	^**^
Palestine	0.053	0.340	0.013	0.821	0.056	0.310	0.072	0.197	0.073	0.186
UK	−0.075	0.182	0.093	0.097	0.017	0.764	0.076	0.172	0.090	0.107
Avoidance of contact with people or their pets: Total	0.037	0.174	0.027	0.323	−0.045	0.100	0.006	0.836	0.118	^**^
Jordan	−0.027	0.615	0.023	0.673	−0.117	0.031	0.104	0.057	0.121	0.026
KSA	−0.024	0.667	0.029	0.598	−0.059	0.285	0.015	0.781	0.200	^**^
Palestine	0.104	0.060	0.012	0.827	0.002	0.972	0.008	0.883	0.120	0.031
UK	0.102	0.067	0.048	0.388	−0.022	0.696	−0.107	0.055	0.031	0.576
Daily habit changes: Total	−0.002	0.952	0.135	^**^	0.011	0.690	−0.009	0.752	0.112	^**^
Jordan	0.033	0.548	0.066	0.226	−0.023	0.675	0.028	0.606	0.034	0.530
KSA	−0.061	0.271	0.194	^**^	−0.068	0.216	−0.091	0.098	0.173	0.002
Palestine	−0.001	0.980	0.122	0.027	0.066	0.231	−0.012	0.830	0.130	0.019
UK	0.037	0.503	0.152	0.006	0.050	0.369	0.042	0.457	0.121	0.031
2. Feeling behavioral changes and distress										
Stress is more: Total	0.415	^**^	−0.087	0.002	0.031	0.267	−0.173	^**^	−0.136	^**^
Jordan	0.357	^**^	−0.180	0.001	−0.003	0.961	−0.062	0.254	−0.117	0.032
KSA	0.370	^**^	0.039	0.484	0.076	0.167	−0.257	^**^	−0.134	0.015
Palestine	0.495	^**^	−0.089	0.110	0.097	0.080	−0.161	0.004	−0.116	0.036
UK	0.447	^**^	−0.129	0.021	−0.095	0.089	−0.221	^**^	−0.184	0.001
Depression is more: Total	0.433	^**^	−0.146	^**^	0.027	0.333	−0.181	^**^	−0.196	^**^
Jordan	0.379	^**^	−0.220	^**^	0.030	0.581	−0.057	0.297	−0.161	0.003
KSA	0.431	^**^	−0.038	0.486	0.068	0.219	−0.279	^**^	−0.186	0.001
Palestine	0.505	^**^	−0.138	0.013	0.078	0.160	−0.171	^**^	−0.161	0.003
UK	0.434	^**^	−0.189	0.001	−0.134	0.016	−0.246	^**^	−0.274	^**^
Sad is more: Total	0.406	^**^	−0.103	^**^	0.006	0.836	−0.179	^**^	−0.166	^**^
Jordan	0.404	^**^	−0.188	0.001	−0.006	0.911	−0.099	0.068	−0.123	0.023
KSA	0.348	^**^	0.006	0.910	0.037	0.498	−0.283	^**^	−0.142	0.010
Palestine	0.460	^**^	−0.114	0.039	0.037	0.499	−0.182	0.001	−0.143	0.010
UK	0.417	^**^	−0.119	0.033	−0.095	0.089	−0.182	0.001	−0.240	^**^
Worry more about the future: Total	0.373	^**^	−0.075	0.007	0.053	0.055	−0.150	^**^	−0.134	^**^
Jordan	0.348	^**^	−0.152	0.005	−0.010	0.852	−0.058	0.289	−0.089	0.100
KSA	0.351	^**^	0.053	0.335	0.070	0.203	−0.221	^**^	−0.153	0.005
Palestine	0.478	^**^	−0.130	0.019	0.095	0.085	−0.202	^**^	−0.149	0.007
UK	0.340	^**^	−0.076	0.171	−0.019	0.735	−0.151	0.007	−0.129	0.020
Feel more lonely after COVID-19: Total	0.319	^**^	−0.080	0.004	0.058	0.035	−0.170	^**^	−0.186	^**^
Jordan	0.305	^**^	−0.179	0.001	0.038	0.486	−0.122	0.025	−0.111	0.042
KSA	0.292	^**^	0.056	0.308	0.079	0.151	−0.219	^**^	−0.233	^**^
Palestine	0.357	^**^	−0.049	0.381	0.092	0.095	−0.192	^**^	−0.127	0.021
UK	0.331	^**^	−0.147	0.008	−0.033	0.556	−0.181	0.001	−0.229	^**^
3. Fears and concerns										
Afraid to get COVID-19: Total	0.218	^**^	0.042	0.123	−0.001	0.957	−0.112	^**^	−0.102	^**^
Jordan	0.127	0.019	−0.023	0.670	0.045	0.407	−0.027	0.617	−0.018	0.744
KSA	0.266	^**^	0.097	0.077	−0.012	0.825	−0.273	^**^	−0.167	0.002
Palestine	0.278	^**^	0.068	0.219	−0.070	0.206	−0.047	0.396	−0.027	0.622
UK	0.199	^**^	0.022	0.690	0.007	0.895	−0.107	0.054	−0.172	0.002
Afraid of lack of healthcare due to COVID-19: Total	0.218	^**^	−0.006	0.819	0.004	0.876	−0.136	^**^	−0.093	0.001
Jordan	0.090	0.097	−0.040	0.461	0.008	0.888	−0.098	0.071	−0.103	0.059
KSA	0.186	0.001	0.092	0.093	0.003	0.957	−0.223	^**^	−0.206	^**^
Palestine	0.347	^**^	−0.040	0.476	−0.015	0.792	−0.158	0.004	0.039	0.485
UK	0.281	^**^	−0.053	0.339	−0.086	0.122	−0.094	0.091	−0.074	0.187
Afraid to lose my job: Total	0.170	^**^	0.008	0.758	0.026	0.349	−0.068	0.014	−0.034	0.222
Jordan	0.079	0.144	−0.009	0.876	0.010	0.858	−0.063	0.249	0.039	0.479
KSA	0.173	0.002	0.049	0.373	−0.025	0.653	−0.142	0.010	−0.139	0.011
Palestine	0.279	^**^	−0.028	0.619	0.011	0.841	−0.118	0.033	−0.026	0.643
UK	0.156	0.005	0.025	0.651	0.053	0.344	0.041	0.460	0.001	0.984
Afraid of separation from my family or beloved ones: Total	0.299	^**^	−0.030	0.281	−0.010	0.713	−0.132	^**^	−0.037	0.175
Jordan	0.201	^**^	−0.091	0.093	−0.007	0.899	−0.010	0.854	−0.038	0.490
KSA	0.311	^**^	0.077	0.160	0.032	0.565	−0.327	^**^	−0.117	0.033
Palestine	0.358	^**^	−0.029	0.607	0.059	0.284	−0.060	0.276	0.002	0.970
UK	0.337	^**^	−0.080	0.150	−0.201	^**^	−0.144	0.010	^**^	0.998
Afraid food and necessary things will not be available: Total	0.272	^**^	0.015	0.596	−0.009	0.751	−0.171	^**^	−0.107	^**^
Jordan	0.259	^**^	−0.037	0.497	0.039	0.474	−0.087	0.111	−0.095	0.081
KSA	0.202	^**^	0.077	0.160	0.016	0.770	−0.236	^**^	−0.212	^**^
Palestine	0.389	^**^	0.023	0.685	−0.024	0.667	−0.123	0.026	−0.008	0.884
UK	0.280	^**^	−0.018	0.743	−0.155	0.005	−0.238	^**^	−0.104	0.063
Worried about contamination of food or water source with COVID-19 virus: Total	0.236	^**^	0.064	0.020	−0.022	0.415	−0.127	^**^	0.013	0.629
Jordan	0.217	^**^	0.041	0.456	0.057	0.294	−0.041	0.447	0.011	0.834
KSA	0.209	^**^	0.182	0.001	0.065	0.241	−0.187	0.001	−0.012	0.824
Palestine	0.239	^**^	−0.004	0.945	−0.033	0.550	−0.066	0.237	0.102	0.065
UK	0.288	^**^	0.033	0.554	−0.181	0.001	−0.191	0.001	−0.073	0.194
Afraid of economic effects on me and my family: Total	0.224	^**^	0.005	0.852	0.055	0.046	−0.088	0.001	0.008	0.770
Jordan	0.153	0.005	0.013	0.806	0.094	0.085	−0.015	0.784	0.019	0.733
KSA	0.262	^**^	0.116	0.036	0.092	0.094	−0.160	0.003	−0.083	0.131
Palestine	0.325	^**^	−0.067	0.228	0.006	0.915	−0.163	0.003	0.094	0.088
UK	0.182	0.001	−0.061	0.278	−0.032	0.573	−0.016	0.773	0.008	0.891
Afraid of punishment if one violates or does not comply with authority measures: Total	0.187	^**^	0.100	^**^	−0.068	0.014	−0.087	0.002	0.032	0.246
Jordan	0.189	^**^	0.056	0.303	−0.049	0.370	−0.050	0.362	0.006	0.907
KSA	0.184	0.001	0.144	0.009	−0.069	0.211	−0.120	0.029	0.058	0.295
Palestine	0.127	0.021	0.123	0.027	−0.059	0.284	−0.064	0.250	0.067	0.225
UK	0.228	^**^	0.092	0.098	−0.033	0.557	−0.093	0.095	−0.008	0.889
Worried about safety of myself and my family: Total	0.256	^**^	−0.001	0.970	0.051	0.066	−0.145	^**^	−0.016	0.555
Jordan	0.262	^**^	−0.052	0.337	0.013	0.816	−0.075	0.167	−0.033	0.545
KSA	0.267	^**^	0.068	0.216	0.104	0.060	−0.262	^**^	−0.094	0.089
Palestine	0.321	^**^	−0.067	0.225	0.061	0.268	−0.123	0.026	0.028	0.619
UK	0.195	^**^	0.030	0.587	−0.051	0.360	−0.134	0.016	0.030	0.586
4. Effects on opinions and beliefs										
Health authorities are not transparent regarding COVID-19: Total	0.199	^**^	−0.069	0.012	0.027	0.321	−0.168	^**^	−0.099	^**^
Jordan	0.189	^**^	−0.088	0.104	−0.016	0.769	−0.156	0.004	−0.111	0.041
KSA	0.168	0.002	−0.033	0.550	0.029	0.596	−0.226	^**^	−0.232	^**^
Palestine	0.269	^**^	−0.150	0.007	0.045	0.415	−0.191	0.001	−0.020	0.721
UK	0.222	^**^	−0.021	0.706	−0.100	0.073	−0.148	0.008	−0.010	0.852
Home self-isolation is acceptable: Total	0.027	0.329	0.059	0.031	−0.011	0.679	0.065	0.018	0.141	^**^
Jordan	0.044	0.418	0.053	0.327	0.023	0.679	0.110	0.043	0.134	0.014
KSA	−0.021	0.707	0.088	0.110	0.032	0.567	0.013	0.812	0.196	^**^
Palestine	0.086	0.122	0.009	0.875	−0.006	0.914	0.120	0.030	0.100	0.071
UK	−0.042	0.448	0.089	0.112	−0.013	0.816	0.025	0.649	0.183	0.001
Imposing curfews is acceptable: Total	0.042	0.127	0.052	0.059	−0.021	0.449	0.055	0.044	0.138	^**^
Jordan	0.079	0.147	0.056	0.302	−0.018	0.739	0.063	0.245	0.135	0.013
KSA	−0.037	0.497	0.053	0.333	0.074	0.181	0.110	0.045	0.229	^**^
Palestine	0.084	0.131	0.057	0.304	−0.005	0.927	0.110	0.046	0.057	0.302
UK	0.006	0.909	0.051	0.357	−0.046	0.414	−0.028	0.613	0.150	0.007
People communication will change after COVID-19: Total	0.084	0.002	0.029	0.288	0.036	0.194	−0.074	0.007	0.007	0.803
Jordan	0.073	0.182	−0.023	0.667	0.019	0.727	0.033	0.540	0.030	0.579
KSA	0.104	0.060	0.128	0.020	−0.014	0.806	−0.223	^**^	−0.116	0.035
Palestine	0.199	^**^	−0.028	0.615	0.024	0.670	−0.178	0.001	0.066	0.231
UK	−0.001	0.983	0.023	0.684	0.046	0.413	0.053	0.340	0.055	0.329

*N, neuroticism; E, extraversion; O, openness; A, agreeableness; C, conscientiousness; r, Spearman Rho correlation coefficient; P, two-tailed probability value*.

** *P <0.0001*.

Moreover, participants from different countries reported different correlations between personality factors and certain COVID-19 impacts (Table 7). For example, Jordanians were the only participants who demonstrated a relationship between neuroticism and VAS scores of washing and disinfecting hands more frequently (*r* = −0.115, *P* = 0.035) (Table 4). Also, they were the only participants who had no significant relationship between neuroticism and fearing the lack of healthcare (*r* = 0.090, *P* = 0.097) or fear of losing jobs (*r* = 0.079, *P* = 0.144) (Table 7). In addition, Jordanians were the only participants who had no relationships between agreeableness and COVID-19 impacts on participants' distress and behaviors (*P* > 0.05) except for the relation between agreeableness and VAS scores for feeling more lonely after COVID-19 (*r* = −0.122, *P* = 0.025) (Table 7). On the other hand, Saudis were the only participants who had no relationships between extraversion and COVID-19 impacts on participants' distress and behaviors (*P* > 0.05) (Table 7). Furthermore, Palestinians were the only participants who had no relationships between openness and different precautions applied by the participants to avoid infection (*P* > 0.05) (Table 7). In addition, Palestinians and Jordanians had no relationships between conscientiousness and any COVID-19-related impacts on participants' fears and concerns (*P* > 0.05) (Table 7). Also, Saudis and Palestinians had no relationships between agreeableness and any CVOID-19 impacts on precautions applied to avoid infection (*P* > 0.05) (Table 7). Moreover, British participants were the only ones who had a significant relationship between openness and participants' distress and behaviors, namely feeling more depressed (*r* = −0.134, *P* = 0.016) (Table 7). Further details of these differences are presented in Table 7.

In order to account for the confounding effects of other demographic factors (including gender, age, level of education, and race), a hierarchical multiple regression analysis was conducted to identify the contribution of personality factors toward (prediction) COVID-19 impacts. It revealed that that personality scores were able to predict VAS scores of COVID-19-related impacts among the total sample as well as within participants from each country (*P* < 0.05) (Table 8). During the hierarchical multiple regression analysis, gender, age, education level, and race variables were included in the first block of independent variables; meanwhile, neuroticism, extraversion, openness, agreeableness, and conscientiousness NEO-FFI dimension scores were included in the second block of independent variables to account for the confounding effects of first block variables on the contribution of NEO-FFI scores toward COVID-19-related factors on VAS scores (i.e., ability of NEO-FFI scores to predict COVID-19-related changes).

**Table 8:** Regression analysis for the contribution of personality factors (prediction) toward COVID-19 impacts among the study population (*N* = 1,319, 744 females and 575 males).

**COVID-19-related impacts**	**1st *R*^**2**^**	**F *R*^**2**^**	**Predictors (*B*, *t*, *P*, 95% CI of *B*) ^$^**
1. Precautions to avoid COVID-19 infection			
Wash and disinfect hands more frequently: Total	0.027	0.053	E (0.062, 4.37, <0.0001, 0.034–0.090), C (0.026, 2.39, 0.017, 0.005–0.048)
Jordan	0.021	0.060	E (0.063, 2.35, 0.020, 0.010–0.116), A (0.080, 2.42, 0.016, 0.015–0.144)
KSA	0.045	0.090	E (0.073, 2.64, 0.009, 0.019–0.127), C (0.043, 2.00, 0.046, 0.001–0.085)
Palestine	0.019	0.054	E (0.061, 2.04, 0.042, 0.002–0.120), C (0.047, 2.06, 0.040, 0.002–0.092)
UK	0.046	0.060	E (0.061, 2.22, 0.027, 0.007–0.114)
Strict social distancing application: Total	0.028	0.037	O (0.028, 2.08, 0.038, 0.002–0.055), C (0.030, 2.83, 0.005, 0.009–0.050)
Jordan	0.024	0.033	No personality predictors
KSA	0.026	0.068	N (0.042, 2.22, 0.027, 0.005–0.079), C (0.077, 3.57, <0.0001, 0.035–0.120)
Palestine	0.012	0.018	No personality predictors
UK	0.052	0.077	E (0.078, 2.95, 0.003, 0.026–0.130)
Crowded area avoidance: Total	0.028	0.043	C (0.037, 4.50, <0.0001, 0.021–0.053)
Jordan	0.025	0.045	C (0.043, 2.66, 0.008, 0.011–0.075)
KSA	0.045	0.101	C (0.070, 4.52, <0.0001, 0.039–0.100)
Palestine	0.021	0.030	No personality predictors
UK	0.030	0.043	E (0.045, 2.08, 0.038, 0.003–0.088)
Avoidance of contact with people or their pets: Total	0.015	0.019	C (0.022, 2.14, 0.033, 0.002–0.043)
Jordan	0.028	0.044	A (0.073, 2.36, 0.019, 0.012–0.134)
KSA	0.042	0.061	C (0.057, 2.57, 0.011, 0.013–0.101)
Palestine	0.029	0.044	No personality predictors
UK	0.007	0.023	No personality predictors
Daily habit changes: Total	0.034	0.047	E (0.070, 4.22, <0.0001, 0.037–0.102)
Jordan	0.016	0.028	No personality predictors
KSA	0.082	0.124	E (0.072, 2.10, 0.036, 0.005–0.140), C (0.066, 2.47, 0.014, 0.013–0.118)
Palestine	0.033	0.053	No personality predictors
UK	0.050	0.067	E (0.075, 2.40, 0.017, 0.014–0.136)
2. Feeling behavioral changes and distress			
Stress is more: Total	0.015	0.174	N (0.193, 14.26, <0.0001, 0.167–0.220), A (−0.062, −2.80, 0.005, −0.105 to −0.019)
Jordan	0.014	0.131	N (0.183, 6.69, <0.0001, 0.129–0.236)
KSA	0.013	0.193	N (0.187, 6.63, <0.0001, 0.132–0.243), E (0.134, 3.28, 0.001, 0.054–0.215), A (−0.170,−3.78, <0.0001, −0.259 to −0.082)
Palestine	0.028	0.246	N (0.249, 9.67, <0.0001, 0.198–0.300)
UK	0.030	0.201	N (0.207, 8.25, <0.0001, 0.157–0.256)
Depression is more: Total	0.039	0.197	N (0.197, 14.48, <0.0001, 0.170–0.223), A (−0.058, −2.62, 0.009, −0.101 to −0.014)
Jordan	0.013	0.151	N (0.187, 6.63, <0.0001, 0.132–0.243), E (0.134, 3.28, 0.001, 0.054–0.215)
KSA	0.022	0.208	N (0.184, 7.15, <0.0001, 0.133–0.234), A (−0.143, −3.31, 0.001, −0.229 to −0.058)
Palestine	0.046	0.256	N (0.253, 9.54, <0.0001, 0.201–0.305)
UK	0.076	0.213	N (0.189, 7.42, <0.0001, 0.139–0.240)
Sad is more: Total	0.024	0.173	N (0.191, 13.73, <0.0001, 0.164–0.218), A (−0.064, −2.81, 0.005, −0.108 to −0.019)
Jordan	0.042	0.170	N (0.201, 7.17, <0.0001, 0.146–0.256)
KSA	0.008	0.172	N (0.176, 6.12, <0.0001, 0.120–0.233), E (0.090, 2.14, 0.033, 0.007–0.172), A (−0.174, −3.78, <0.0001, −0.264 to −0.083)
Palestine	0.031	0.217	N (0.236, 8.74, <0.0001, 0.183–0.289)
UK	0.035	0.182	N (0.193, 7.55, <0.0001, 0.143–0.244)
Worry more about the future: Total	0.021	0.144	N (0.186, 13.74, <0.0001, 0.160–0.213)
Jordan	0.031	0.129	N (0.170, 6.14, <0.0001, 0.115–0.224)
KSA	0.034	0.185	N (0.200, 6.76, <0.0001, 0.142–0.258), E (0.145, 3.37, 0.001, 0.060–0.229), A (−0.103, −2.18, 0.030, −0.196 to −0.010)
Palestine	0.016	0.221	N (0.250, 9.19, <0.0001, 0.197–0.308)
UK	0.021	0.118	N (0.152, 5.91, <0.0001, 0.101–0.202)
Feel more lonely after COVID-19: Total	0.032	0.140	N (0.162, 11.29, <0.0001, 0.134–0.190), A (−0.062, −2.65, 0.008, −0.107 to −0.016)
Jordan	0.024	0.114	N (0.163, 5.81, <0.0001, 0.108–0.219)
KSA	0.031	0.190	N (0.168, 5.85, <0.0001, 0.111–0.224), E (0.155, 3.62, <0.0001, 0.071–0.240), A (−0.103, −2.25, 0.025, −0.194 to −0.013), C (−0.093, −2.87, 0.004, −0.157 to −0.029)
Palestine	0.011	0.149	N (0.203, 7.22, <0.0001, 0.147–0.258)
UK	0.050	0.136	N (0.158, 5.63, <0.0001, 0.103–0.213)
3. Fears and concerns			
Afraid to get COVID-19: Total	0.002	0.060	N (0.115, 7.60, <0.0001, 0.085–0.144), E (0.088, 4.13, <0.0001, 0.046–0.130), A (−0.065, −2.72, 0.007, −0.112 to −0.018)
Jordan	0.004	0.020	N (0.068, 2.32, 0.021, 0.010–0.126)
KSA	0.043	0.162	N (0.135, 4.73, <0.0001, 0.079–0.192), E (0.136, 3.27, 0.001, 0.054–0.218), A (−0.164, −3.60, <0.0001, −0.254 to −0.074)
Palestine	0.018	0.104	N (0.156, 5.21, <0.0001, 0.097–0.214), E (0.104, 2.39, 0.018, 0.018–0.189), O (−0.091, −2.18, 0.030, −0.173 to −0.009)
UK	0.004	0.048	N (0.103, 3.80, <0.0001, 0.050–0.156)
Afraid of lack of healthcare due to COVID-19: Total	0.014	0.079	N (0.121, 7.55, <0.0001, 0.090–0.153), E (0.064, 2.84, 0.005, 0.020–0.109), A (−0.103, −4.07, <0.0001, −0.153 to −0.054)
Jordan	0.002	0.018	N (0.074, 2.36, 0.019, 0.012–0.135)
KSA	0.004	0.139	N (0.100, 3.13, 0.002, 0.037–0.162), E (0.198, 4.14, <0.0001, 0.104–0.292), A (−0.162, −3.17, 0.002, −0.262 to −0.061), C (−0.133, −3.69, <0.0001, −0.204 to −0.062)
Palestine	0.041	0.163	N (0.170, 5.56, <0.0001, 0.110–0.230), A (−0.117, −2.27, 0.024, −0.219 to −0.016)
UK	0.003	0.080	N (0.143, 5.16, <0.0001, 0.088–0.198)
Afraid to lose my job: Total	0.012	0.054	N (0.123, 7.68, <0.0001, 0.092–0.154), E (0.052, 2.25, 0.024, 0.007–0.097)
Jordan	0.035	0.051	N (0.074, 2.38, 0.018, 0.013–0.136)
KSA	0.024	0.064	N (0.110, 3.74, <0.0001, 0.052–0.168)
Palestine	0.006	0.092	N (0.177, 5.55, <0.0001, 0.114–0.239)
UK	0.033	0.071	N (0.106, 3.59, <0.0001, 0.048–0.164)
Afraid of separation from my family or beloved ones: Total	0.024	0.100	N (0.149, 9.44, <0.0001, 0.118–0.180), E (0.053, 2.36, 0.018, 0.009–0.097), A (−0.053, −2.12, 0.034, −0.102 to −0.004)
Jordan	0.029	0.062	N (0.110, 3.45, 0.001, 0.047–0.172)
KSA	0.019	0.177	N (0.167, 5.33, <0.0001, 0.105–0.228), E (0.149, 3.27, 0.001, 0.059–0.238), A (−0.225, −4.50, <0.0001, −0.323 to −0.126)
Palestine	0.051	0.150	N (0.171, 6.12, <0.0001, 0.116–0.226)
UK	0.043	0.146	N (0.184, 6.19, <0.0001, 0.126–0.243), C (0.094, 2.92, 0.004, 0.031–0.158)
Afraid food and necessary things will not be available: Total	0.030	0.107	N (0.134, 8.83, <0.0001, 0.104–0.163), E (0.092, 4.30, <0.0001, 0.050–0.134), A (−0.087, −3.63, <0.0001, −0.134 to −0.040)
Jordan	0.051	0.102	N (0.125, 4.33, <0.0001, 0.068–0.181)
KSA	0.032	0.135	N (0.101, 3.19, 0.002, 0.039–0.163), E (0.163, 3.45, 0.001, 0.070–0.256), A (−0.146, −2.88, 0.004, −0.245 to −0.046), C (−0.097, −2.71, 0.007, −0.167 to −0.026)
Palestine	0.025	0.160	N (0.201, 7.14, <0.0001, 0.145–0.256), E (0.095, 2.30, 0.022, 0.014–0.177)
UK	0.076	0.156	N (0.102, 3.69, <0.0001, 0.048–0.157), E (0.111, 2.73, 0.007, 0.031–0.191), A (−0.122, −2.86, 0.005, −0.205 to −0.038)
Worried about contamination of food or water source with COVID-19 virus: Total	0.025	0.084	N (0.112, 7.64, <0.0001, 0.083–0.140), E (0.108, 5.24, <0.0001, 0.068–0.149), A (−0.060, −2.58, 0.010, −0.105 to −0.014)
Jordan	0.039	0.070	N (0.091, 3.33, 0.001, 0.037–0.145)
KSA	0.009	0.104	N (0.141, 4.73, <0.0001, 0.084–0.198), E (0.204, 4.73, <0.0001, 0.119–0.288)
Palestine	0.006	0.074	N (0.125, 4.57, <0.0001, 0.071–0.179), C (0.089, 2.86, 0.005, 0.028–0.150)
UK	0.081	0.159	N (0.112, 3.83, <0.0001, 0.054–0.169), E (0.151, 3.53, <0.0001, 0.067–0.235), A (−0.102, −2.28, 0.023, −0.189 to −0.014)
Afraid of economic effects on me and my family: Total	0.008	0.067	N (0.122, 9.13, <0.0001, 0.096–0.148), E (0.044, 2.31, 0.021, 0.007–0.082)
Jordan	0.004	0.063	N (0.093, 3.76, <0.0001, 0.044–0.142), O (0.100, 2.69, 0.007, 0.027–0.173)
KSA	0.016	0.127	N (0.177, 6.07, <0.0001, 0.120–0.234), E (0.174, 4.03, <0.0001, 0.089–0.259)
Palestine	0.036	0.175	N (0.152, 5.82, <0.0001, 0.101–0.204), A (−0.116, −2.72, 0.007, −0.200 to −0.032), C (0.073, 2.57, 0.011, 0.017–0.130)
UK	0.011	0.040	N (0.072, 3.13, 0.002, 0.027–0.117)
Afraid of punishment if one violates or does not comply with authority measures: Total	0.034	0.085	N (0.115, 7.79, <0.0001, 0.086–0.144), E (0.113, 5.28, <0.0001, 0.071–0.155), O (−0.053, −2.63, 0.009, −0.093 to −0.014)
Jordan	0.027	0.055	N (0.093, 3.12, 0.002, 0.034–0.151)
KSA	0.019	0.092	N (0.124, 4.27, <0.0001, 0.067–0.182), E (0.159, 3.65, <0.0001, 0.074–0.245), O (−0.094, −2.13, 0.034, −0.180 to −0.007)
Palestine	0.054	0.082	N (0.076, 2.67, 0.008, 0.020–0.132), E (0.088, 2.11, 0.035, 0.006–0.170)
UK	0.016	0.086	N (0.135, 4.53, <0.0001, 0.076–0.194), E (0.139, 3.16, 0.002, 0.052–0.226)
Worried about safety of myself and my family: Total	0.019	0.081	N (0.114, 7.97, <0.0001, 0.086–0.142), E (0.063, 3.11, 0.002, 0.023–0.102), A (−0.067, −2.97, 0.003, −0.112 to −0.023)
Jordan	0.032	0.079	N (0.111, 4.13, <0.0001, 0.058–0.165)
KSA	0.009	0.123	N (0.135, 4.51, <0.0001, 0.076–0.194), E (0.110, 2.54, 0.012, 0.025–0.196), A (−0.170, −3.57, <0.0001, −0.264 to −0.076)
Palestine	0.043	0.140	N (0.162, 6.01, <0.0001, 0.109–0.215), C (0.066, 2.15, 0.032, 0.006–0.126)
UK	0.015	0.057	N (0.091, 3.52, <0.0001, 0.040–0.141), E (0.090, 2.38, 0.018, 0.016–0.165)
4. Effects on opinions and beliefs			
Afraid health authorities are not transparent regarding COVID-19: Total	0.035	0.088	N (0.097, 6.41, <0.0001, 0.067–0.126), A (−0.095, −3.89, <0.0001, −0.144 to −0.047)
Jordan	0.024	0.085	N (0.141, 4.73, <0.0001, 0.083–0.200)
KSA	0.028	0.105	N (0.062, 2.06, 0.040, 0.003–0.122), A (−0.153, −3.04, 0.003, −0.252 to −0.054), C (−0.075, −2.19, 0.029, −0.143 to −0.008)
Palestine	0.048	0.128	N (0.111, 3.68, <0.0001, 0.051–0.170), A (−0.141, −2.78, 0.006, −0.241 to −0.041)
UK	0.014	0.054	N (0.097, 3.64, <0.0001, 0.044–0.149)
Home self-isolation is acceptable: Total	0.023	0.038	A (0.047, 3.15, 0.002, 0.018–0.076), C (0.027, 2.64, 0.008, 0.007–0.047)
Jordan	0.023	0.039	A (0.081, 2.38, 0.018, 0.014–0.148)
KSA	0.033	0.056	C (0.043, 2.79, 0.006, 0.013–0.074)
Palestine	0.016	0.048	A (0.099, 3.33, 0.001, 0.040–0.157)
UK	0.030	0.048	No personality predictors
Imposing curfews is acceptable: Total	0.023	0.038	A (0.044, 2.75, 0.006, 0.013–0.075), C (0.033, 3.07, 0.002, 0.012–0.055)
Jordan	0.023	0.035	No personality predictors
KSA	0.013	0.055	C (0.074, 3.82, <0.0001, 0.036–0.112)
Palestine	0.015	0.038	A (0.095, 2.78, 0.006, 0.028–0.162)
UK	0.023	0.035	E (0.061, 2.00, 0.046, 0.001–0.120)
People communication will change after COVID-19: Total	0.034	0.054	N (0.054, 3.75, <0.0001, 0.026–0.082), A (−0.057, −2.45, 0.014, −0.103 to −0.011)
Jordan	0.012	0.025	N (0.059, 2.06, 0.040, 0.003–0.114)
KSA	0.047	0.118	N (0.080, 2.70, 0.007, 0.022–0.138), E (0.136, 3.16, 0.002, 0.051–0.221), A (−0.149, −3.15, 0.002, −0.242 to −0.056)
Palestine	0.013	0.078	N (0.074, 2.62, 0.009, 0.019–0.130), A (−0.148, −3.09, 0.002, −0.243 to −0.054)
UK	0.062	0.070	No personality predictors

*N, neuroticism; E, extraversion; O, openness; A, agreeableness; C, conscientiousness; 1st R^2^, 1st block R^2^ (coefficient of determination) using confounding variables (covariates) gender, age, education, and ethnicity in the first block of the hierarchical multiple regression analysis; F R^2^, final R^2^ (coefficient of determination) after the addition of personality factors in the second block of the hierarchical multiple regression analysis; B, beta statistics; t, t statistics; P, probability value; 95% CI of B, confidence intervals of beta*.

$ *Personality factors (predictors) that contribute toward COVID-19-related impacts identified using hierarchical multiple regression analysis. The significance of F statistic change for all statements was P <0.05*.

The multiple regression analysis showed that conscientiousness and extraversion were the major personality factors that predict COVID-19 impacts on participants' applied precautions to avoid COVID-19 infection (*P* < 0.05) (Table 8). Higher conscientiousness and extraversion scores were associated with more acceptance and application of applied precautions to avoid COVID-19 infection. On the other hand, neuroticism and agreeableness were the major personality factors that predict COVID-19 impacts on participants' distress and behaviors (*P* < 0.05) (Table 8). Higher neuroticism and lower agreeableness scores were associated with more distress and negative behaviors due to COVID-19. Also, neuroticism, extraversion, and agreeableness were the major personality factors that predict COVID-19 impacts on participants' concerns and fears (*P* < 0.05) (Table 8). Higher neuroticism, lower extraversion, and lower agreeableness were associated with more concerns and fears due to COVID-19. Finally, neuroticism and agreeableness were the major personality factors that predict COVID-19 impacts on participants' opinions and beliefs. Higher neuroticism and lower agreeableness were associated with more impacts of COVID-19 on opinions and beliefs.

It is interesting to find that openness was the least personality factor involved in prediction toward different COVID-19 impacts. It was only involved in the prediction of VAS scores for application of strict social distancing among the total study population (*B* = 0.028, *t* = 2.08, *P* = 0.038, 95% CI = 0.002–0.055), fear of COVID-19 infection among Palestinians (*B* = −0.091, *t* = −2.18, *P* = 0.030, 95% CI = −0.173 to −0.009), fear of economic effects of COVID-19 among Jordanians (*B* = 0.100, *t* = 2.69, *P* = 0.007, 95% CI = 0.027–0.173), and fear of punishment if one violates authority measures among the total study populations (*B* = −0.053, *t* = −2.63, *P* = 0.009, 95% CI = −0.093 to −0.014) and Saudis (*B* = −0.094, *t* = −2.13, *P* = 0.034, 95% CI = −0.180 to −0.007) (Table 8). Details of the contribution of personality factors toward COVID-19 impacts among the total study population and among participants from each country are presented in Table 8.

Generally, higher neuroticism scores were associated with more COVID-19-related concerns, worries, fears, and negative impacts (*P* < 0.05) (Tables 7, 8). However, higher extraversion, openness, agreeableness, and conscientiousness scores were associated with more acceptance of COVID-19 containment measures as well as less COVID-19-related concerns, worries, fears, and negative impacts (*P* < 0.05) (Tables 7, 8).

## Discussion

This study showed that COVID-19 was associated with changes and impacts on precautions to avoid infection, distress and behavioral changes, fears and concerns, and effects on opinions and beliefs. Also, significant relationships were identified between personality factors and COVID-19-related impacts. Therefore, the null hypothesis was rejected.

The NEO-FFI test was used in this study because it permits a comprehensive measurement of personality dimensions following the Five-Factor Model of Personality (34–45), and it is simple, reliable, valid, and sensitive (34, 35, 38, 40, 44, 45). It was used in previous studies among Jordanian, British, Saudi, and Palestinian populations (35–42, 44).

Information regarding personality scores prior to COVID-19 is not available due to the unexpected fast spreading outbreak of the COVID-19 pandemic. It is important to notice that personality factors are stable and they might take a long time to change (46). Further prospective exploration is required in this regard.

In this study, COVID-19 was associated with changes in people's daily activities, habits, opinions, distress, concerns, fears, and worries regarding many aspects of life including personal health and hygiene; economy issues and work; social and physical distancing; availability of healthcare, resources, and supplies; exercise and outdoor activities; psychological effects; and communication with others. This concur the findings of other studies that demonstrated the presence of pressures, stressors, and concerns due to COVID-19 including worries regarding infection, personal health, health of family and loved ones, work pressures and changes, shortage of supplies and resources, ability to control the disease, economy and financial issues, stigma, social distancing, and isolation (2, 4–6, 18, 19, 47, 48).

Also, this concurs the findings of previous studies that showed changes in health behavior and application of preventive measures including hand hygiene, physical distancing, social distancing, altered daily activities, and avoidance of contacts and gatherings (4, 5, 18, 19, 47).

The outcomes of this research showed that COVID-19 was associated with feelings of stress, depression, sadness, and loneliness. This agrees with previous findings that reported higher levels of stress, anxiety, depression, mental problems, and PSTD following the COVID-19 pandemic (4, 5, 13, 18, 19, 47, 49, 50). In addition, reduced levels of happiness and satisfaction with life were reported following the COVID-19 outbreak, and individuals were more worried regarding their health and families and less worried regarding friends and leisure (13).

The negative impacts of COVID-19 on daily habits as well as mental and psychological well-being might be attributed to concerns regarding incomplete understanding of the pathogenesis and transmission of COVID-19, lack of undisputed treatments for the virus, uncertainty in dealing with the disease during the early stages of the pandemic, imposing quarantine and curfews on large scales, the increase in the number of COVID-19-positive or suspected cases, and overloading the media with rumors and information regarding serious deficiencies in medical staff, hospital capacities, supplies, and resources (5, 10, 19).

Also, it was reported that higher levels of psychological distress were strongly correlated with fear of getting infected with COVID-19, better knowledge regarding the COVID-19 pandemic, and fears concerning job and future of profession (14, 48).

The results of this investigation demonstrated that females reported differences in some COVID-19-related impacts, concerns, fears, and behaviors than males. These differences might be attributed to the variations in the underlying personality factors between genders.

This concurs the findings of previous studies that reported higher negative psychological impacts of COVID-19 on females and younger individuals (8, 11, 19). Also, previous research demonstrated that females reported more psychological problems and had more anxiety, distress, and depression than males (11, 18, 19, 50). However, some researchers demonstrated no differences between genders regarding generalized anxiety disorder during the COVID-19 pandemic (4). Furthermore, some researchers found that dysfunctional personality factors and casual beliefs rather than gender or age were behind reported emotional well-being problems during the early stages of COVID-19 in Italy (12). Differences from these studies might be attributed to variations in personality factors, cultural backgrounds, and duration and severity of COVID-19 outbreak and associated containment measures.

In this study, relationships were established between personality factors and COVID-19-related impacts, fears, concerns, and behaviors. Generally, higher neuroticism scores were associated with more COVID-19-related concerns, worries, fears, and negative impacts. However, higher extraversion, openness, agreeableness, and conscientiousness scores were associated with more acceptance of COVID-19 containment measures as well as less COVID-19-related concerns, worries, fears, and negative impacts. Different personality dimensions contributed to the prediction of COVID-19-associated impacts, fears, concerns, and behaviors.

These findings are in agreement with previous conclusions that less negative impacts on well-being were associated with higher openness and conscientiousness in the USA (29). Meanwhile, more negative impacts on daily living were associated with higher neuroticism during the pandemic in Germany (30).

This is also in line with the previous findings that higher neuroticism was associated with more pessimistic duration estimates, more concerns, and fewer precautions in response to COVID-19 in the USA (25), better hygiene and social distancing in the USA (24), and better commitment to social distancing and application of governmental measures against COVID-19 in Qatar (31). In addition, lower neuroticism was associated with better commitment to apply prescribed COVID-19 instructions (27), as well as improved sanitation and better social distancing (26) in the USA. Also, lower levels of extraversion were accompanied with better hygiene and social distancing in the USA (24), better commitment to social distancing and application of governmental measures against COVID-19 in Japan (33), and acknowledgment of the importance of handwashing and social distancing to combat COVID-19 in Brazil (23).

Moreover, higher extraversion was associated with more optimistic duration estimates (25), better commitment to apply prescribed COVID-19 instructions (27), and better social distancing, sanitation, and handwashing (28) in the USA. Also, higher openness was associated with better commitment to social distancing and application of governmental measures against COVID-19 in Japan (33), and improved sanitation and better social distancing (26), better commitment to apply prescribed COVID-19 instructions (27), and better hygiene practice (24) in the USA.

Furthermore, higher agreeableness was associated with better commitment to apply prescribed COVID-19 instructions in the USA (27), better hygiene and social distancing in the USA (24), and better commitment to social distancing and application of governmental measures against COVID-19 in Germany (32) and Japan (33).

Finally, higher levels of conscientiousness were accompanied with more precautions (25), better commitment to apply prescribed COVID-19 instructions (27), better sanitation and handwashing (28), and better hygiene and social distancing (24) in the USA. Higher levels of conscientiousness were also accompanied with acknowledgment of the importance of handwashing and social distancing as containment measures against COVID-19 in Brazil (23), as well as better commitment to social distancing and application of governmental measures against COVID-19 in Qatar (31) and Japan (33).

However, this contrasts with previous findings that anxiety had no significant relationships with behavior changes or measures used by the public to prevent infection among a Chinese population (18). This difference might be attributed to different cultural backgrounds; differences in demographic variables; the use of different measures to evaluate psychological factors, severity, and spread of the outbreak; and the difference in time being affected with COVID-19 and containment measures (2).

The current study included and compared participants from different countries. Nevertheless, the literature lack previous research to compare different countries in this regard. The interaction between personality factors and COVID-19-related impacts revealed some variations between the participants from different countries in this study. This might be attributed to different cultural backgrounds, the variations in personality factors, and the differences in disease impacts, spread, and severity (2).

The above discussion demonstrated that COVID-19 has various impacts on humanity including the psychological and mental well-being of people from different backgrounds. It is important to note that different individuals might react differently to the same issues related to the COVID-19 outbreak as has been demonstrated by the findings of this study. Therefore, individual differences and personality factors might contribute to the explanation of the differences in the response to COVID-19 and associated impacts, fears, concerns, and behaviors. In addition, disease burden and associated issues might potentially impact mental well-being and personality factors especially if it existed for a longer duration, and further research is required in this regard.

The findings of this study give an insight regarding the potential commitment with different disaster containment measures applied by authorities in order to find ways to improve acceptance and application of these measures among the population. This information might be included in mitigation plans set to combat infectious outbreaks during COVID-19 as well as potential future disease outbreaks. After all, containment of the effects of different dangers depends on the response and interactions of individuals to the applied measures.

In this regard, measuring personality and mental well-being is potentially useful to indicate the level of commitment to containment measures and the severity of impacts caused by the disease on various life aspects within different communities. Therefore, authorities could include promoting mental health and protection against mental illness and personality problems within their mitigation plans to avoid the severe effects of infectious outbreaks and associated containment measures.

In addition, this should be promoted through current interventions as well as future universal plans to reduce the negative impacts of current and future infectious disease outbreaks on individuals and communities. Special attention could be observed when communities exhibit higher levels of neuroticism and lower levels of extraversion, openness, agreeableness, and conscientiousness, because these factors could be potentially accompanied with more negative impacts of infectious disease outbreaks on individuals and communities. Provision of psychological counseling and plans to reduce the burden of containment measures could help in this regard, and this requires further investigations.

Further prospective longitudinal studies are required to evaluate changes in concerns, behaviors, fears, and daily habits due to COVID-19. Also, further assessment of personality and psychological impacts of COVID-19 is required using adequate comprehensive tests of personality and psychology among different populations. In addition, further long-term cohort studies are required to identify long-term psychological well-being of individuals from different backgrounds.

### Study Strengths and Limitations

The current study sample is very diverse covering four countries, namely three Arab Middle-Eastern samples from Jordan, Saudi Arabia, and Palestine and the UK. In addition to the total study sample, the data was also analyzed within each of the four separate samples. Therefore, the study provided valuable cross-cultural information about the associations between FFM personality dimensions and their combinations and interactions with COVID-19-related concerns and behaviors.

In addition, this study employed a comprehensive and extremely well-regarded and validated personality inventory (the NEO Five-Factor Inventory) which is a shortened version (60 items) of the 240-item NEO Personality Inventory-Revised which is considered the gold-standard measure of the Big Five or Five-Factor Model of Personality. Also, the relationships between personality factors and various concerns, fears, impacts, and behaviors related to COVID-19 were investigated in this study.

However, the conduction of this study was associated with some limitations. First, the cross-sectional design of the study with concealed participants' identity has limited the possibility to generalize the cause–effect relationship between the COVID-19 outbreak and changes in personality factors. However, the study mainly aimed to study the relationship between personality factors and COVID-19-related impacts, concerns, fears, and behaviors rather than simply to evaluate the changes in personality factors due to COVID-19. In addition, the confounding effects of various demographic variables on the relationships between NEO-FFI scores and COVID-19 impact scores were accounted for and considered during the statistical analysis of this study. Second, this study was based on an online survey to avoid cross infection, and this might affect sampling as people who are interested might have been more willing to participate than those who are not interested. However, the study was multicentered and participants from different centers were invited more than once *via* email and then *via* different social media platforms to ensure it reaches all potential participants. Third, the personality factors of participants before this pandemic were not available for comparisons with personality factors following COVID-19. Therefore, existing personality factors might have been present even before COVID-19. However, this investigation aimed to identify the relationship between personality factors and COVID-19-related impacts, concerns, fears, and behaviors rather than merely to assess changes in personality factors.

## Conclusions

In conclusion and within the limitations of this study, it was concluded that COVID-19 was associated with impacts on participants' applied precautions to avoid COVID-19 infection, distress and behavioral changes, fears and concerns, and effects on opinions and beliefs.

Personality factors were associated with and able to predict COVID-19-related impacts on applied precautions to avoid infection, distress and behavioral changes, fears and concerns, and effects on opinions and beliefs. Higher neuroticism scores were associated with more COVID-19-related concerns, distress, worries, fears, and negative impacts on daily life and habits. Meanwhile, higher extraversion, openness, agreeableness, and conscientiousness scores were associated with more acceptance of COVID-19 containment measures (such as social distancing and handwashing and disinfection) as well as less COVID-19-related concerns, worries, fears, and negative impacts on daily life and habits.

## Data Availability Statement

The raw data supporting the conclusions of this article will be made available by the authors, without undue reservation.

## Ethics Statement

The studies involving human participants were reviewed and approved by the Institutional Review Board, University of Jordan, Amman 11942, Jordan (Reference number: 223/2020/19/31). The patients/participants provided their written informed consent to participate in this study.

## Author Contributions

MA-O, AKA, and MM conceived the study. MA-O, MM, IA, AKA, and AAA designed the study, collected the data, interpreted the data, and drafted sections of the manuscript. MA-O and MM prepared the survey tool for the study. MA-O carried out the data analysis and critically revised the manuscript. EL contributed to data collection and critically revised the manuscript. All authors read and approved the submitted final version of the manuscript, and agree to be accountable for the content of the work.

## Conflict of Interest

The authors declare that the research was conducted in the absence of any commercial or financial relationships that could be construed as a potential conflict of interest.
